# Thioredoxin-mimetic peptide CB3 lowers MAPKinase activity in the Zucker rat brain^☆^

**DOI:** 10.1016/j.redox.2013.12.018

**Published:** 2014-01-09

**Authors:** Moshe Cohen-Kutner, Lena Khomsky, Michael Trus, Hila Ben-Yehuda, James M. Lenhard, Yin Liang, Tonya Martin, Daphne Atlas

**Affiliations:** a Department of Biological Chemistry, Institute of Life Sciences, The Hebrew University of Jerusalem, Jerusalem 91904 Israel; b Cardiovascular and Metabolic Research, Janssen Research & Development, LLC of Johnson and Johnson, Welsh and McKean Roads, Springhouse, PA 19477, USA

**Keywords:** Ad-AMPK-CA, AMPK-constitutively active AMP-activated protein kinase mutants, AICAR, 5-amino-4-imidazole carboxamide riboside, AMPK, AMP-activated protein kinase, TXNIP/TBP-2, thioredoxin-interacting protein, CB3, NAc-Cys-Pro Cys-amide, TXM-CB3, Diabetes type 2, Inflammation, Thioredoxin mimetics, ZDF rat-model, MAPK, AMPK, TXNIP/TBP-2, CB3, Oxidative stress, Redox

## Abstract

Diabetes is a high risk factor for dementia. High glucose may be a risk factor for dementia even among persons without diabetes, and in transgenic animals it has been shown to cause a potentiation of indices that are pre-symptomatic of Alzheimer's disease. To further elucidate the underlying mechanisms linking inflammatory events elicited in the brain during oxidative stress and diabetes, we monitored the activation of mitogen-activated kinsase (MAPKs), c-jun NH_2_-terminal kinase (JNK), p38 MAP kinases (p38^MAPK^), and extracellular activating kinsae1/2 (ERK1/2) and the anti-inflammatory effects of the thioredoxin mimetic (TxM) peptides, Ac-Cys-Pro-Cys-amide (CB3) and Ac-Cys-Gly-Pro-Cys-amide (CB4) in the brain of male leptin-receptor-deficient Zucker diabetic fatty (ZDF) rats and human neuroblastoma SH-SY5Y cells. Daily i.p. injection of CB3 to ZDF rats inhibited the phosphorylation of JNK and p38^MAPK^, and prevented the expression of thioredoxin-interacting-protein (TXNIP/TBP-2) in ZDF rat brain. Although plasma glucose/insulin remained high, CB3 also increased the phosphorylation of AMP-ribose activating kinase (AMPK) and inhibited p70^S6K^ kinase in the brain. Both CB3 and CB4 reversed apoptosis induced by inhibiting thioredoxin reductase as monitored by decreasing caspase 3 cleavage and PARP dissociation in SH-SY5Y cells. The decrease in JNK and p38^MAPK^ activity in the absence of a change in plasma glucose implies a decrease in oxidative or neuroinflammatory stress in the ZDF rat brain. CB3 not only attenuated MAPK phosphorylation and activated AMPK in the brain, but it also diminished apoptotic markers, most likely acting via the MAPK–AMPK–mTOR pathway. These results were correlated with CB3 and CB4 inhibiting inflammation progression and protection from oxidative stress induced apoptosis in human neuronal cells. We suggest that by attenuating neuro-inflammatory processes in the brain Trx1 mimetic peptides could become beneficial for preventing neurological disorders associated with diabetes.

## Highlights

• Thioredoxin mimeitics peptides (TXM) lower apoptosis in the brain of ZDF rat.
• TxM peptides prevent TXNIP/TBP-2 expression in the brain of ZDF rat.
• TxM peptides could become beneficial for preventing diabetes associated neurological disorders.

## Graphical abstract

**Figure:**
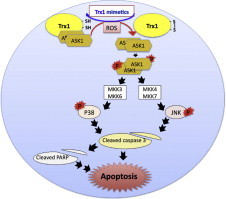

## Introduction

Aging patients with Type 2 diabetes (T2D) are at a high risk of developing cognitive and memory impairments including some of Alzheimer disease's (AD) most significant symptoms [1]. In recent years it has become evident that some characteristics of AD are regulated by insulin-like growth factor signaling cascades [2]. The greatest risk factor of AD and T2D is age and one of the major hallmarks of the aging process is oxidative stress. The thioredoxin reductase–thioredoxin system (TrxR–Trx1) is part of the powerful enzymatic machinery that maintains the redox balance of the cell [3], [4]. Neuronal Trx1 is decreased in AD brains and Trx1 is oxidized by the β-amyloid (Aβ) peptide, through an inflammatory mediated apoptotic cycle. Trx1 regulates apoptosis by inhibiting the apoptosis signal-regulating kinase-1 (ASK1), which activates the JNK and p38^MAPK^ pathways [5]. Trx1 also prevents apoptosis through association with other proteins like the Trx1-interacting protein-2 (TBP-2) also called TXNIP or VDUP-1. While TXNIP/TBP-2 binds to the active Cys residue of Trx1 and inhibits its redox activity, Trx1 itself binds the non-catalytic region of ASK1 and inhibits its kinase activity [6], [7], [8], [9], [10], [11], [12], [13]. TXNIP/TBP-2 is a member of early response genes involved in neuronal apoptosis induced by high glucose, oxidative stress, or Ca^2+^. It was shown to regulate the transcription factor c-jun in cerebellar granule neurons [14]. Neuronal cell death induced by ischemic–reperfusion or hyperglycemic–ischemic–reperfusion was prevented by the down regulation of TXNIP/TBP-2 [15]. The divergent effects of glucose and fatty acids on TXNIP/TBP-2 expression result in part from their opposing effects on AMP-activated protein kinase (AMPK) activity. The effects of high glucose on insulin resistance, which have been attributed to insulin receptor substrate phosphorylation, are induced through a decrease in AMPK, a heterotrimeric protein composed of a catalytic subunit (α) and two regulatory subunits (β and γ) that are activated in anaerobic conditions [16], [17]. Activation of the AMPK pathway by metformin treatment normalized impaired cell proliferation and neuroblast differentiation in the subgranular zone of the hippocampal dentate gyrus in Zucker diabetic fatty (ZDF) rats [18]. High-glucose levels in the lateral hypothalamus also decreased the expression of the AMPK gene [19]. More recently it was demonstrated that activation of AMPK alleviates high glucose-induced dysfunction of brain microvascular endothelial cells by suppressing the induction of NADPH oxidase-derived superoxide anions [20]. The loss of islet DNA binding activity of pancreas duodenum homeobox-1 and insulin gene expression in the ZDF rat was prevented in animals treated with troglitazone [21], or N-acetyl cysteine (NAC) [22]. Since NAC has antioxidant activity, it was hypothesized that glucose toxicity in the ZDF animal may be explained in part by chronic oxidative stress [23]. Furthermore, JNK activity, which was elevated by oxidative stress causing β-cell dysfunction, was overcome by suppression of the JNK pathway [24]. In liver, muscle and adipose tissues of dietary and genetic (*ob*/*ob*) obesity models, there was a significant increase in total JNK activity, highlighting JNK as a crucial mediator of obesity and insulin resistance, and a potential target for therapeutics [25]. In the ovalbumin (OVA)-inhaled mice, a rodent model of asthma, treatment with NAc-Cys-Pro Cys-amide (CB3), a thioredoxin mimetic peptide [26], [27], prevented reactive oxygen species (ROS) related damages through inhibition of p38^MAPK^ activation and prevention of NF-kB nuclear translocation [28]. In the present study we explored CB3 ability to protect the brain from multiple factors involved in the oxidative stress pathway associated with diabetes. We showed that the Trx1 mimetic peptides CB3 known to inhibit JNK and p38^MAPK^ phosphorylation in fibroblasts [29], neuroendorine PC12 [26], and INS 832/13 insulinoma cells [27], prevented apoptosis in human neuroblastoma SH-SY5Y cells. We show that in the ZDF rat brain, CB3 lowered markers of inflammation, reduced TXNIP/TBP-2 expression, activated AMPK and thereby inhibited the mTOR–p70^S6K^ pathway. Hence, CB3 could have a potential benefit for decreasing detrimentaleffects elicited in the brain during chronic hyperglycemia.

## Materials and methods

### Reagents

All materials were purchased from Sigma, Jerusalem, if not otherwise stated; Auranofin (Enzo life sciences, Shoham, Israel), triethylphosphine (2,3,4,6-tetra-O-acetyl-β-1-d-thiopyranosato-S) gold(I); thioredoxin mimetic (TXM) peptides TXM-CB3 and -CB4 were custom synthesized by Novetide, Ltd. Haifa; Thinkpeptides, Oxford, UK, and GL Biochem., Shanghai, China; tissue culture serum and medium were from Biological Industries, Kibbutz Beit-Haemek, Israel.

### Cells

SH-SY5Y human neuroblastoma cells were kindly provided by H Soreq H. (Hebrew University of Jerusalem, Israel). The cells were cultured in DMEM/F12 HAM 1:1 medium supplemented with 10% fetal bovine serum (FBS) and penicillin–streptomycin, incubated at 37 °C with 5% CO_2._

### Cell viability

SH-SY5Y cells were seeded in 96-well plates and treated with 5 µM AuF for 30 min, or high glucose, washed and cultured with or without increasing concentrations of CB3, or CB4, as indicated. Twenty-four hours later, the cells were fixed with glutaraldehyde in a final concentration of 0.5% for 10 min. Cells were washed 3 times with DDW, dried over night, and washed once with borate buffer (0.1 M, pH 8.5). The fixed cells were stained with 200 µl of 1% methylene blue in borate buffer for 1 h. After extensive washing and drying, the color was extracted with 200 µl of 0.1 M HCl for 1 h at 37 °C. Later cell viability was measured using spectrophotometer at 630 nm.

### Zucker diabetic fatty (ZDF) rat animal study

In this study we used the obese diabetic Zucker rat, a widely used animal model of obesity and type 2 diabetes. These animals display insulin resistance, dyslipidemia, hyperinsulinemia [30], [31] and, in some colonies, hypertension develops by 4–5 months of age [32].

Six-weeks old male ZDF rats were injected with either CB3 (1 mg/kg and 10 mg/kg) or with Rosiglidasone (Rosi) for 28 days. Blood glucose was measured every week (glucometer). At day 26 an OGTT assay was obtained and measured. At day 28, the animals were sacrificed and different biochemical blood markers (Table 1) were measured. Animal brains were collected, homogenized and quantified for protein content. The samples from these animals were separated by SDS-PAGE and analyzed by western blot as described.

**Table 1:** Weekly analysis of blood glucose levels, OGTT measurement at day 26, HbA1c blood levels, triglyceride blood levels, insulin blood levels and NEFAs blood levels at day 28.

**Parameter**	**Saline (Zucker)**	**CB3**, 1 **mg/kg**	**CB3**, **10** **mg/kg**	**Rosi**, **10** **mg/kg**
**HbA1c (%)**	7.24±0.56	7.49±0.51	9.54±0.85	4.56±0.05*
**Triglyceride (mg/dl)**	873.5±51.4	881.7±40.7	1065.0±147.8	112.4±7.07*
**NEFAs (mEq/L)**	0.61±0.06	0.58±0.06	0.47±0.08	0.14±0.03*
**Insulin (ng/ml)**	13.5±2.9	18.6±2.9	13.4±2.6	6.5±2.1
**MCP-1 (pg/ml)**	6845±587	5532±834	5361±786	5307±874

The values shown are the averages (±SEM) of all animals in each group. Student's *t*-test (two populations) was performed for ZDF rats treated with saline only (Zucker).

⁎ *P* value<0.05; (*n*=4–8).

### Western blot analysis

Twenty to thirty micrograms of protein samples were loaded on 10–12% SDS-PAGE gels. The proteins were then transferred electrophoretically to nitrocellulose (Whatman, Germany). The blots were blocked by incubation for 1 h at RT in TBS-T (25 mM Tris–HCl pH 7.4, 0.9% NaCl and 0.02% Tween-20) with 4% Difco skim milk (BD, USA), and incubated over-night at 4 °C with the primary antibody: pERK1/2 (Thr 202/Tyr204), mouse mAb; ERK2 (Santa Cruz, U.S.A) rabbit Ab; p-SAPK/JNK (Thr183/Tyr185), rabbit mAb; SAPK/JNK, mouse mAb; p-p38MAP kinase (Thr180/Tyr182), rabbit mAb; p38, rabbit Ab; cleaved caspase 3, rabbit mAb; PARP (Poly (ADP-ribose) polymerase), rabbit Ab; GAPDH (glyceraldehyde 3-phosphate dehydrogenase), rabbit mAb; TXNIP/TBP-2 mouse mAb. Antibodies were from Cell Signaling Tech. USA, if not otherwise stated, used at 1:1000. Purified b Catenin, mouse mAb, (1:10,000; BD Transduction Laboratories, USA) diluted in 5% BSA, 0.04% azide in TBS-T. Proteins were detected with anti-mouse or anti-rabbit IgG-HRP linked antibody (1:10,000; Cell Signaling, Tech. USA). For data analysis, the amounts of each band were quantified by using the EZ-Quant software (version 2.2) and plotted with a linear regression program.

## Results

### CB3 had no effect on blood glucose or insulin content levels

We used ZDF rats characterized by a progressive β-cell dysfunction and a leptin receptor defect, which result in hyperglycemia. The ZDF rats were divided into 4 groups. Animals were i.p. injected with vehicle (0.9% saline), 1 mg/kg CB3, 10 mg/kg CB3, or p.o. with 10 mg/kg rosiglitazone (Rosi), an antidiabetic agent, which activates peroxisome proliferator-activated receptor gamma (PPAR-γ agonist). Blood glucose levels and plasma insulin were tested as indicated (Table 1). Rats treated with Rosi displayed a significant decrease in blood glucose and plasma insulin levels compared to the control group. In contrast, CB3 had no effect on either blood glucose or plasma insulin in the groups compared to the saline supplemented animals (Table 1).

### CB3 lowered MAPK JNK and p38 phosphorylation, but not MAPK ERK1/2 in brain of ZDF rats

**Fig. 1:**
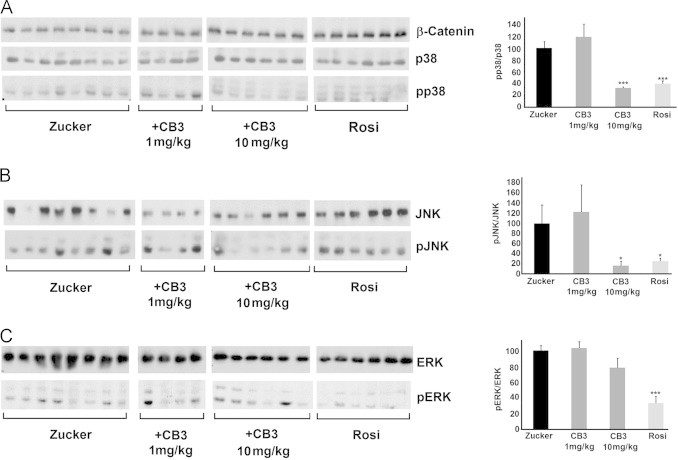
CB3 inactivates JNK and p38 but not ERK1/2 in the brains of ZDF rats. ZDF rats were supplemented with either CB3 or Rosi for 28 days (as described in Table 1). Brain samples of each animal from each group were homogenized and proteins were separated by SDS-PAGE (Section 2). The blots were incubated with antibodies against (**A**) p38^MAPK^ phospho-p38^MAPK^ and β-catenin (B) JNK and phospho-JNK or (**C**) ERK1/2 and phospho-ERK 1/2. Each band represents a single animal of each group. The values were quantified shown as the averages (±SEM) of all the bands presented in the blots (right). The values were normalized to the phosphorylation state of ZDF rats treated with saline only (Zucker). Student's *t* test (two populations) was performed for ZDF rats treated with saline only (Zucker). ^*^P value<0.05; ^**^P value<0.01; and ^***^P value<0.005, (*n*=4–8).

To explore whether CB3 protected the ZDF rat brain from the effects of high glucose, we monitored the inflammatory state of the brain analyzing the phosphorylation level of three kinases, c-Jun NH_2_-terminal kinase (JNK), p38 MAP kinase (p38^MAPK^) and the extracellular-signal-regulated kinases 1 and 2 (ERK1/2). Rats injected with 1 mg/kg of CB3 showed no significant change in p38^MAPK^, JNK, or ERK1/2 phosphorylation compared to the untreated group. In contrast, the phosphorylation level of both p38^MAPK^ and JNK was significantly lowered in animals treated with 10 mg/kg CB3 or with 10 mg/kg Rosi (Fig. 1A and B). The reduction by CB3 suggests a specific effect of the Trx1 mimetics, which by increasing the ratio of Trx1_re_/Trx1_ox_, prevented Trx1–ASK1-dissociation, and inhibited the Trx1–ASK1–MAPK pathway [27]. The significant decrease in JNK and p38^MAPK^ phosphorylations in the Rosi-treated rats most probably was secondary to PPARγ-mediated changes in metabolism.

ERK1/2 is activated by intracellular accumulation of free radicals and involves another inflammatory cascade [33], [34], independent of the ASK1–Trx1 pathway. Therefore it was expected to be less sensitive to CB3. Indeed, no significant reduction in ERK1/2 phosphorylation was observed in the CB3 treated animals (Fig. 1C). Given Rosi reduced glucose in plasma and CB3 did not, these data suggest that the changes in ERK1/2 may be secondary to altered fuel metabolism.

### The TxM-mimetics, CB3 and CB4, prevent MAPK induction by blocking thioredoxin reductase or by TNFα

**Fig. 2:**
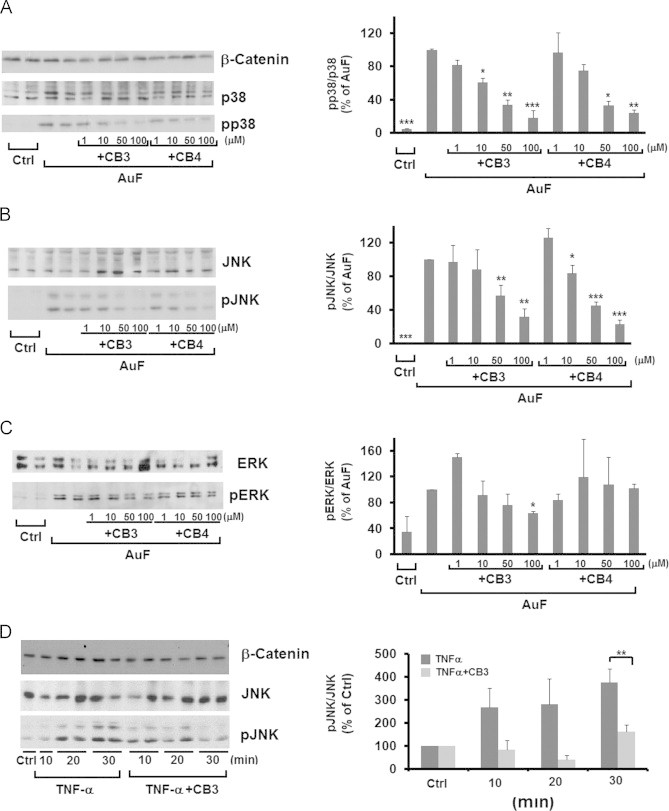
CB3 and CB4 reverse the phosphorylation of JNK and p38^MAPK^ but not ERK1/2 in SH-SH5Y cells. SH-SY5Y cells were treated with 5 µM AuF for 30 min, washed, and treated with or without increasing concentrations of CB3 and CB4, as indicated. Cell lysates were separated by SDS-PAGE and the phosphorylation of (A) JNK (B) p38^MAPK^ or (C) ERK1/2 were visualized by immunoblots using the appropriate antibodies (see above) and quantified (right). The values are averages (±SEM) of three independent experiments normalized to the phosphorylation state of cells treated with AuF. (D) Cells treated with 5 ng/ml TNF-α, with or without CB3 (100 µM) at the indicated time intervals. Equal amounts of whole-cell lysates were separated on SDS-PAGE and JNK phosphorylation was determined by immunoblots (left) and quantified (right). The values are averages (±SEM) of three independent experiments normalized to control cells. Student's *t* test (two populations) was performed for AuF/TNF-a treated cells. ^*^P value<0.05; ^**^P value<0.01; and ^***^P value<0.005.

We next examined the consequences of CB3 on inflammatory pathways induced in SH-SY5Y cells, a human neuroblastoma cell line often used as a cellular model of AD. In addition we used CB4, another member of the thioredoxin-mimetic family TxM-CB4 (NAc-Cys-Gly-Pro-Cys amide), which was previously shown to be effective in reversing amyloid beta-induced protein oxidation, loss of mitochondrial function and DNA fragmentation in primary neuronal cells [29]. CB4 was also effective in reversing oxidaitve stress-induced apoptosis in PC12 [26], and insulinoma cells [27]. We monitored p38^MAPK^ and JNK phosphorylation/activation induced by exposure of the cells to auranofin (AuF), a potent TrxR inhibitor. By keeping Trx1 in the oxidized-state, AuF leads to the dissociation of oxidized Trx1 from ASK1, activating the ASK1–MAPK cascade [5]. SH-SY5Y cells were treated for 30 min with 5 µM AuF, washed and incubated for 2 h with or without CB3 or CB4 at the indicated concentrations. The phosphorylation of MAPK was monitored by western blot analysis using selective antibodies against phosphorylated p38^MAPK^, JNK, and ERK1/2, and the corresponding non-phosphorylated MAPKs (Fig. 2A, B and C). The reduction of AuF-induced JNK and p38^MAPK^ phosphorylation was concentration-dependent (Fig. 2A and B). CB3 and CB4 were considerably more effective in reducing AuF-induced JNK and p38^MAPK^ phosphorylation (Fig. 2A and B) compared to the AuF-induced ERK1/2 phosphorylation (Fig. 2C). This result is consistent with the lack of any significant effect of CB3 on ERK1/2 phosphorylation in the ZDF brain (Fig. 2C). This specific inhibition of JNK and p38^MAPK^ phosphorylation by TxM, further supports the view that the Trx1 mimetics act through preventing ASK1–Trx1 dissociation

Further evidences for the anti inflammatory effects of the TxM peptides were achieved by examining TNFα, a ROS-independent inflammatory reagent known as a JNK activator [35]. SH-SY5Y cells were exposed to 5 ng/ml TNFα with or without CB3 (100 µM) for 10, 20 and 30 min. At these time intervals JNK activation was significantly reduced by CB3, further supporting the anti-inflammatory effects of CB3 (Fig. 2D).

### CB3 reduces TXNIP/TBP-2 levels in the brain of ZDF rats

**Fig. 3:**
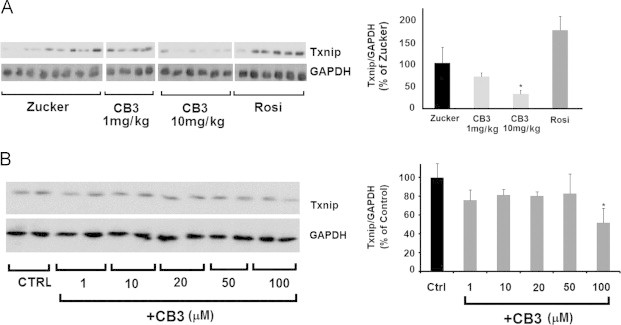
CB3 reduces TXNIP/TBP-2 levels in the brain of ZDF rats and in SH-SY5Y cells. ZDF rats were supplemented with either CB3 or Rosi for 28 days as indicated in Fig. 1. Brain samples were lysed and proteins were separated on SDS-PAGE (A) left, TXNIP/TBP-2, levels were determined using TXNIP/TBP-2 antibodies using anti GAPDH antibodies as a reference. Right, all values of each group were collected and normalized to GAPDH. (B) SH-SY5Y cells were exposed to increasing concentrations of CB3, as indicated. The level of TXNIP/TBP-2 was determined using anti TXNIP antibodies (left), and the data was quantified using GAPDH as a reference (right). The results represent the averages (±SEM) of all the bands presented in the blots. All values were normalized to the TXNIP/TBP-2 levels of ZDF rats treated with saline only (Zucker) or to the levels of control cells. Student's *t* test (two populations) was performed for ZDF rats treated with saline only (Zucker) or to control cells. ^*^P value<0.05; ^**^P value<0.01; and ^⁎⁎⁎^*P* value<0.005, (*n*=4–8).

Next we explored the expression and the impact of CB3 on the expression of TXNIP/TBP-2 in the ZDF rat. As shown in Fig. 3A, a significant reduction in TXNIP expression was observed in the brain of animals treated with 10 mg/kg of CB3, but not with 1 mg/kg. In contrast, in the Rosi-treated rats no significant reduction in TXNIP/TBP-2 expression was observed, in spite of a strong reduction in blood glucose. These results suggest that the Trx mimetic peptide most probably lowers an intrinsically high level of TXNIP/TBP-2 in the ZDF rats independent of blood glucose. Further studies are required to explore the nature of the glucose dependency of the elevated levels of TXNIP/TBP-2 in the ZDF rat brain.

Unlike the high glucose up-regulation of TXNIP/TBP-2 in beta cells [36], high glucose in neuronal SH-SY5Y cells had no apparent effect on TXNIP/TBP-2 expression (data not shown). CB3 (100 µM) appeared to cause a substantial reduction in the constitutive TXNIP/TBP-2 expression in these cells (Fig. 3B).

### Adenosine-mono phosphate (AMP) activated protein kinase (AMPK) is activated in the brain of CB3 treated ZDF rats

**Fig. 4:**
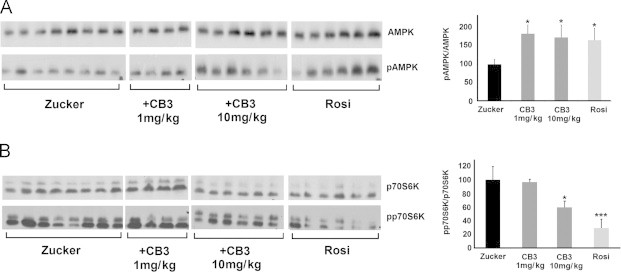
CB3 increases AMPK activation and inhibits p70S6 kinase in the brains of ZDF rats. ZDF rat brain samples were separated by SDS-PAGE as described. The blots of each group, were incubated with antibodies against (A) AMPK, and pAMPK and (B) p70S6K, and phospho p70S6K. Each band represents a single animal in each group. The data was quantified (right) represent averages (±SEM) of three independent experiments. The values were normalized to the ZDF rats treated with saline only (Zucker). Student's *t* test (two populations) was performed for ZDF rats treated with saline only (Zucker). ^*^P value<0.05; ^**^P value<0.01; and ^***^P value<0.005, (*n*=4–8).

The anti-diabetic drugs, Rosi and metformin are known as activators of the AMPK pathway, which reduce intracellular ATP by inhibiting complex I of the mitochondrial electron transport chain [37]. Therefore, we measured the AMPK alpha Thr172 phosphorylation in the brain of ZDF rats that were treated with 10 mg/kg Rosi, 1 mg/kg, and 10 mg/kg of CB3. As expected, Rosi-treated animals showed almost a two-fold increase in AMPK activation (Fig. 4A). Surprisingly, AMPK was equally activated in the brain of 1 or 10 mg/kg of CB3 injected ZDF rats.

The phosphorylation level of AMPK, which leads to inhibition of the mammalian target of rapamycin (m-TOR) pathway, was further evaluated in the ZDF brain. AMPK mediates m-TOR inhibition through binding of Raptor and phosphorylation of p70S6 kinase, a protein involved in numerous cell-signaling pathways. We observed that in both CB3 and Rosi treated animals phosphorylation of p70S6 kinase in the ZDF brain was reduced (Fig. 4B). These results suggest that AMPK activation by CB3 led to the inhibition of the downstream AMPK–m-TOR-signaling, similar to the effect of Rosi.

### CB3 and CB4 protect SH-SY5Y cells from AuF toxicity

**Fig. 5:**
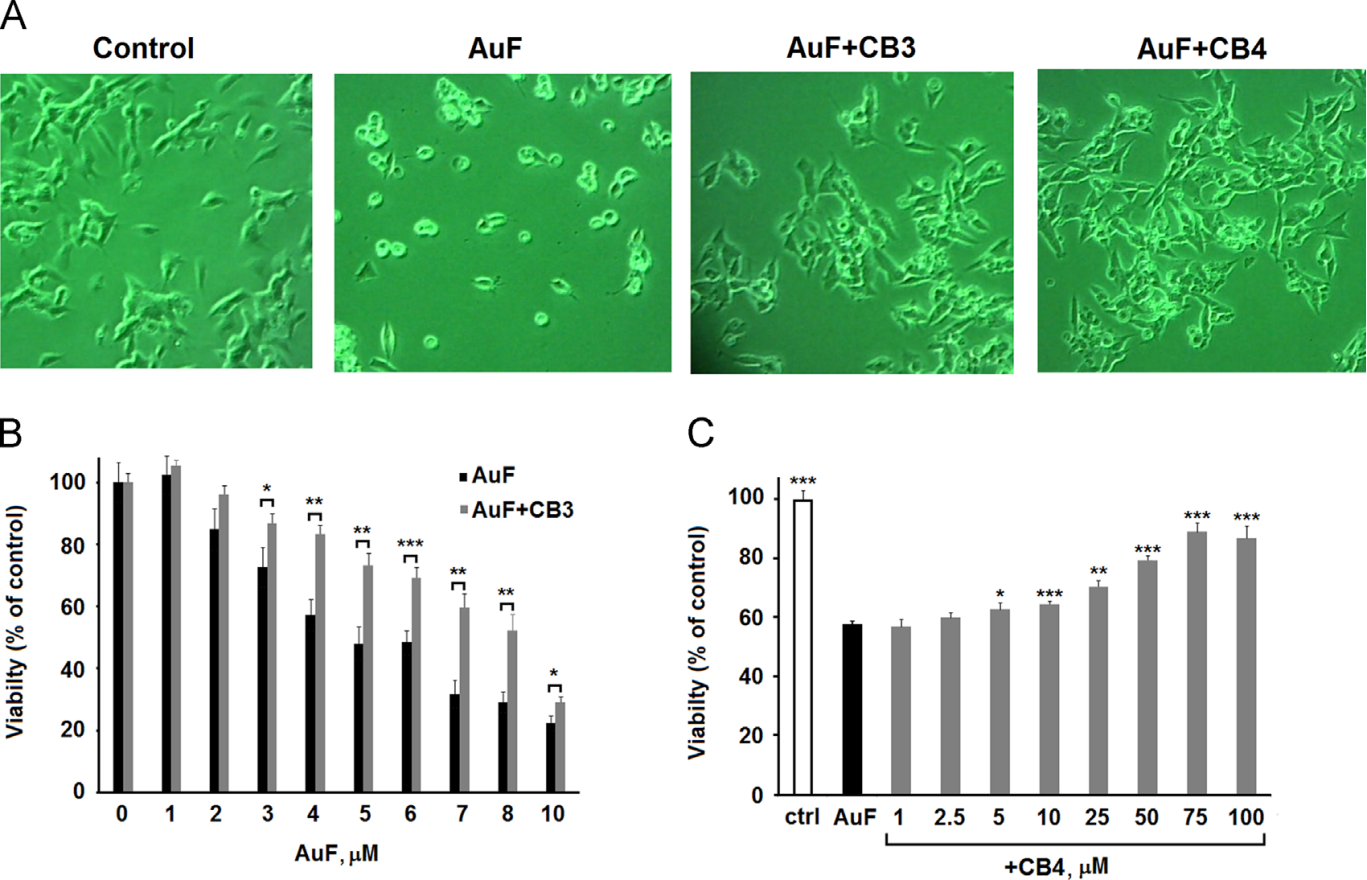
TXM peptides -CxC- and -CxxC- protect SH-SH5Y cells from AuF-induced cell death. (A) Phase-microscope images of SH-SY5Y cells treated with AuF and with CB3 or CB4, taken after 24 h (magnification, ×100). (B) The cells were incubated with increasing concentrations of AuF for 30 min, washed and incubated with or without CB3 (100 µM). The cells were tested for viability using the methylene blue assay after 24 h (C) Viability of cells pre-treated with 5 µM AuF, washed and later exposed to increasing concentrations of CB4, was determined 24 h later. Data is displayed as mean±S.E.M (*n*=8–12). Student's *t* test (two populations) was performed for AuF treated cells. ^*^P value<0.05; ^**^P value<0.01; and ^***^P value<0.005.

The effects of AuF on cell viability and the protection offered by CB3 and CB4 were visualized and quantified in SH-SY5Y cells. The cells were treated with AuF (5 µM) for 30 min, washed, and visualized 24 h later. Phase contrast microscopy demonstrated a considerable change in cell morphology and cell number (Fig. 5A). In contrast, most of the CB3- or CB4-treated cells appeared healthy under phase-contrast microscopy, showing normal shape and well-developed cell to-cell contact (Fig. 5A). The decrease in cell viability by AuF (1–10 µM) was quantified using the methylene blue viability assay (see Section 2) [27]. After 24 h the number of viable cells was significantly increased in the presence of 100 µM CB3 at all AuF concentrations (Fig. 5B). Rescue from 5 µM AuF toxicity was also seen in cells treated with CB4 in a concentration dependent manner (Fig. 5C).

### CB3 and CB4 inhibit caspase 3 and PARP dissociation in SH-SY5Y cells

**Fig. 6:**
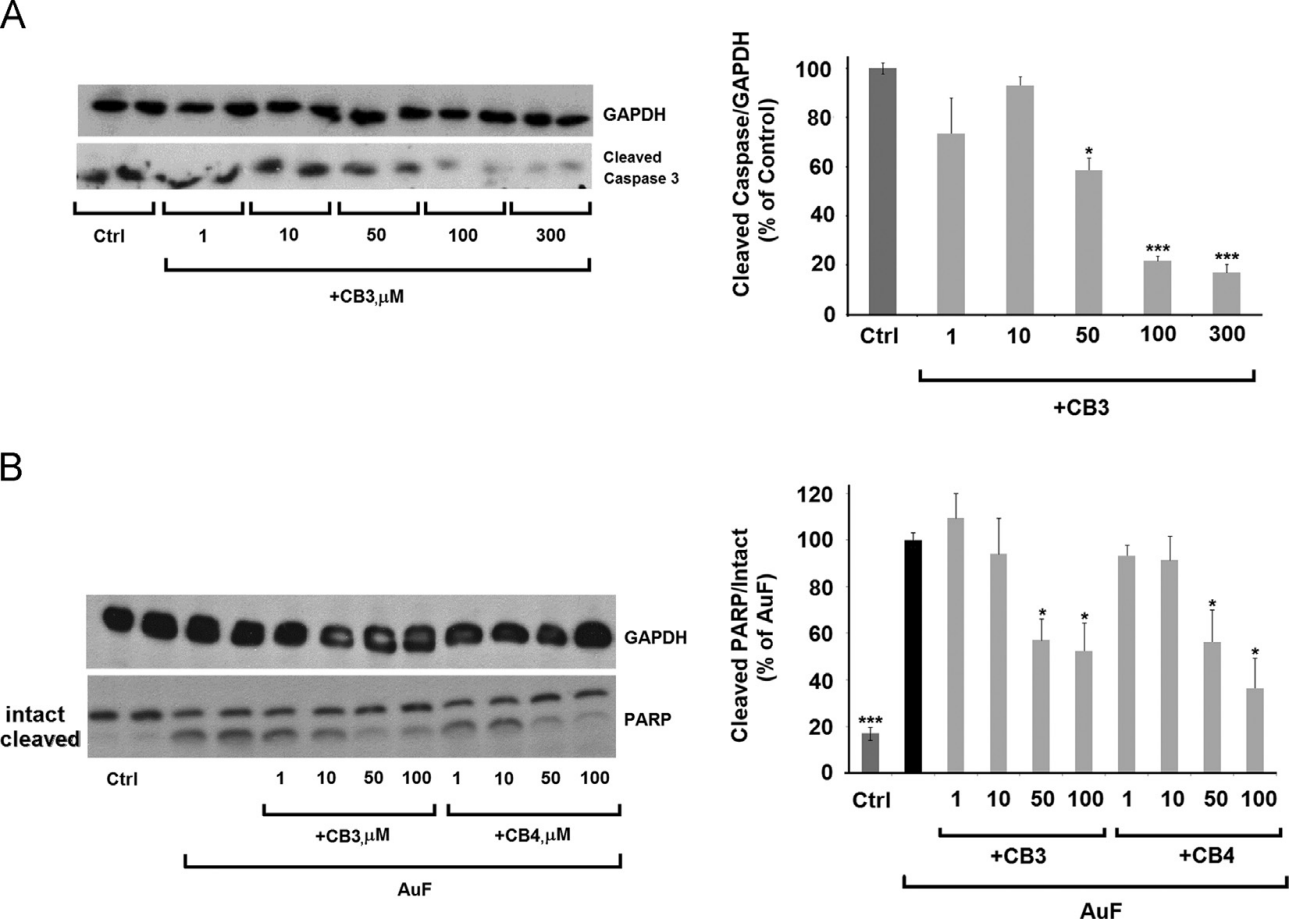
CB3 and CB4 inhibit caspase 3 and PARP dissociation in SH-SY5Y cells. (A) SH-SY5Y **c**ells were treated for 24 h with or without CB3 at the concentrations as indicated. Equal proteins of whole-cell lysates were separated by SDS-PAGE. Caspase 3 cleavage was detected using antibodies against cleaved caspase-3. (B) Increasing concentrations of CB3 or CB4 were tested for preventing AuF-induced PARP dissociation. PARP dissociation was detected using antibodies against PARP. The values were quantified as shown (right) are averages (±SEM) of three independent experiments. Student's *t* test (two populations) was performed for either control or AuF treated cells in B. ^*^P value<0.05; and ^***^P value<0.005.

Next we tested the effect of CB3 on caspase 3-cleavage in SH-SY5Y cells. The cells were incubated with 100 µM CB3 for 24 h in serum-free medium. A reduction in caspase 3-cleavage was observed in CB3 treated cells in a concentration dependent manner, seen already at 50 µM (Fig. 6A). We then examined the nuclear enzyme poly (ADP-ribose) polymerase (PARP), which is constitutively expressed in the cell and stimulated allosterically by DNA single-strand breaks that are generated during a redox injury [38]. During apoptosis PARP is dissociated by caspase 3 and loses its activity to induce necrosis [30]. Treatment with 5 µM AuF increased PARP dissociation consistent with the viability assays (Fig. 5). A considerable decrease in PARP dissociation was observed in AuF-treated cells that were exposed to CB3 or CB4 (Fig. 6B). These results further confirm the anti-apoptotic properties of TxM peptides [26], [27].

## Discussion

In this study we analyzed the protection of ZDF rat brain and human SH-5Y5Y neuroblastoma cells from oxidative induced inflammation damages and from inflammatory consequences accompanying diabetes or through disruption of the TrxR–Trx redox system. For this purpose we used the thioredoxin mimetic peptides, CB3 and CB4. These peptides derived from the canonical CxxC motif of the Trx1 active site and a modified CxC motif, which are responsible for the redox activity of Trx1.

### CB3 inhibits MAPK phosphorylation in ZDF rat brain

The TxM thiol peptides alleviate oxidative stress by inhibiting JNK and p38^MAPK^ phosphorylations and preventing NF-kB nuclear translocation in vitro and in vivo [26], [27], [28], [29]. It was shown that obesity increases cerebrocortical ROS and impairs brain function [39]. Diabetes is also a significant risk factor for dementia in general, including AD, and probably vascular dementia [40]. Dietary fat intake was shown in epidemiological studies to increase the risk of incident dementia [41] and decrease Morris maze performance [42]. This further confirms the role of high glucose in destructing brain function. The anti-inflammatory and anti-apoptotic properties of TxM peptides could prove to be useful in relieving oxidative stress elicited in the brain of obese rats, which led us to test CB3 in the ZDF brain.

Here we tested inhibition by CB3 of inflammatory pathways that are activated by MAP-Kinases, JNK and p38, in the ZDF rat brain. Although no changes in blood glucose were observed, the CB3 treated mice displayed a decrease in the phosphorylation/activation of the MAPK inflammatory-stress pathway with its ensuing apoptotic effects. Although the decrease in phosphorylated JNK and 38^MAPK^ in the brain might indicate that CB3 crosses the blood brain barrier (BBB) in order to protect against inflammatory neurodegenerative consequences in the ZDF rats, more direct studies are required to establish BBB penetration of TxM peptides.

Interestingly, in previous studies N-acetyl cysteine (NAC), which is a much weaker reducing reagent compared to CB3 [26], resulted in a significant reduction in blood glucose of the ZDF rat [22], [43]. The decrease in plasma glucose by NAC, which became apparent at the 9th week [22], [43] suggest that to ascertain reduction in blood glucose it would be important to monitor blood glucose in CB3-treated ZDF rats over a longer period compared to the present study [22]. The lower level of MAPK phosphorylation in the Rosi-treated rats could be attributed in part, to its ability to prevent glucose increase, or to a PPAR-specific effect. Rosi was demonstrated to attenuate endotoxin lethality by inhibiting HMGB1 release in a mouse model of sepsis [18].

In studies carried out using insulinoma cells, CB3 appeared to prevent apoptosis through inhibiting the Trx1–ASK1–MAPK pathway [27]. Protection of the ZDF rat brain from the inflammatory damage is consistent with TXM antiapoptotic affects seen also in the neuroendocrine PC12 [26] and insulinoma cells [27].

### TxM-peptides rescue SH-5HSY cells from apoptosis

**Fig. 7:**
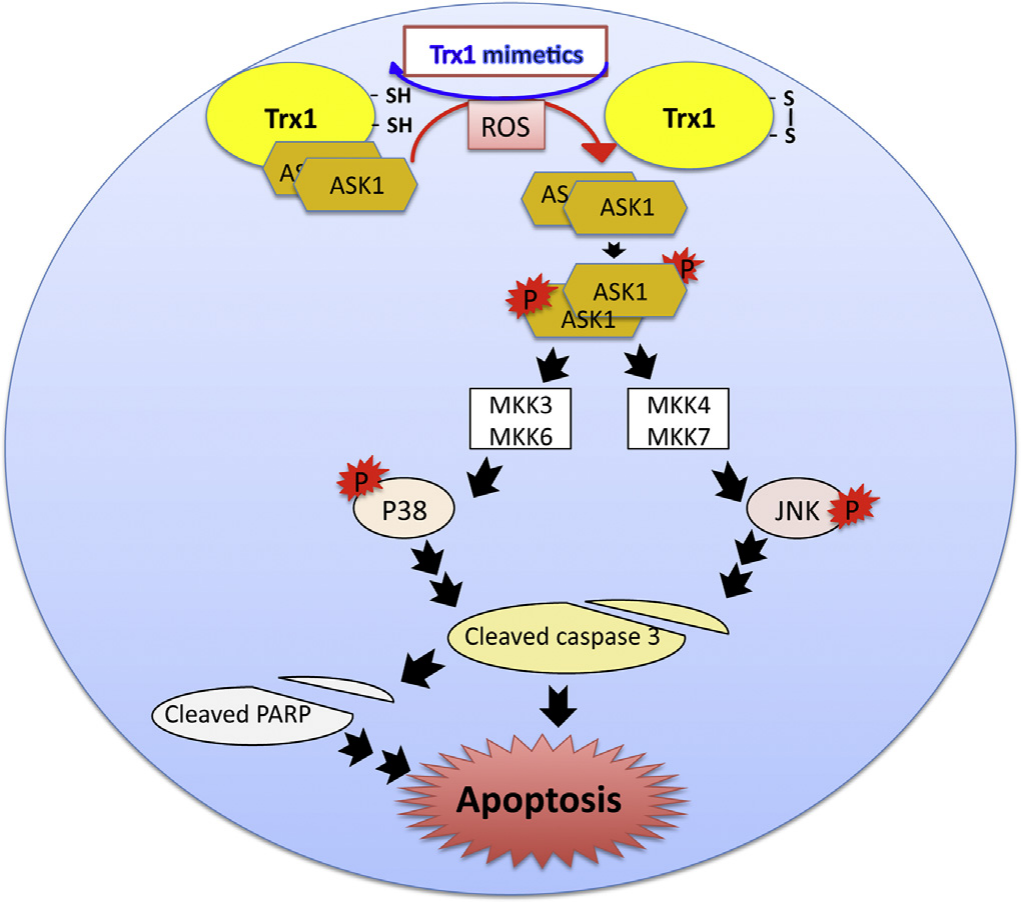
Schematic presentation of Trx1 mimetic peptides acting to reverse ASK1–MAPK signaling induced by ROS/glucose in the ZDF rat brain.

Human neuronal SH-5Y5Y cells are often considered a model for Alzheimer's disease. These cells, when treated with CB3 or CB4, displayed protection from oxidative stress induced by blocking the [TrxR–Trx] redox system. The increase in cell viability, which was accompanied by a decrease in caspase-3 cleavage, prevention of PARP dissociation, as well as the ability to reverse TNF-alpha induced JNK phosphorylation in SH-SH5Y cells, further supports the anti-inflammatory properties of these peptides. TxM putative activity pathway is shown schematically in Fig. 7. Consistent with the in vivo ZDF data, these results suggest that inhibiting the TRX–ASK1–MAPK pathway, which is accompanied by an increase in AMPK, could protect rat brain neuronal cells from apoptosis and implicate a potential use of this Trx1 mimetic peptide for treating inflammation induced by high glucose. The in vivo and in vitro data is consistent with TXM proposed activity previously shown using insulinoma 832/13 cells [27].

### CB3 lowers TXNNI/TBP-2 expression in ZDF rat brain

TXNIP/TBP-2 is a key stress-responsive inhibitory switch of Trx1 activity playing an important role in the preservation of cellular viability [44]. Recent knockout studies, suggested that inhibition of TXNIP/TBP-2, up regulates both insulin sensitivity and glucose-stimulated insulin secretion in diabetes, and might present a novel therapeutic approach for T2DM [13], [45]. Also in humans, TXNIP/TBP-2 was shown to regulate peripheral glucose [46].

We observed a significant decrease in TXNIP/TBP-2 levels in CB3 treated ZDF rats. The mechanism by which CB3 lowers TXNIP/TBP-2 currently remains unknown. It is possible that by lowering ROS, CB3 prevents TXNIP/TBP-2 up regulation through inhibiting transcription. This possibility is consistent with a recent study demonstrating that TXNIP/TBP-2 expression in the brain was induced by oxidative stress without glucose [15]. Consistent with the results of Trx1 over expression, which was shown to be neuroprotective against ischemic brain damage [47], the Trx1 mimetic CB3 appeared to dramatically prevent oxidative stress damages by lowering MAP kinase activity as well as TXNIP/TBP-2 expression in the ZDF brain. Alternatively, by reducing the disulfide bridge between Cys32/Cys35 and TXNIP/TBP-2, CB3 induces TXNIP/TBP-2 dissociation from Trx1. The Trx1-free-TXNIP/TBP-2 in turn, inhibits TXNIP transcription, down regulating the transcriptionally activated carbohydrate response element-binding protein.

In the Rosi-treated animals, in which glucose and triglycerides levels were low, TXNIP/TBP-2 level was not decreased. In contrast, in CB3-treated animals in which glucose and triglycerides levels were high, altering of the Trx/TXNIP redox balance, CB3 appeared to regulate TXNIP/TBP-2 in a glucose independent mechanism.

Unlike a strong induction of TXNIP/TBP-2 by high glucose in insulinoma cells [48], the level of TXNIP/TBP2 in SH-SY5Y cells was constitutively high and was not induced further by high glucose. The TXNIP/TBP-2 level, which was lowered by CB3 and CB4, could result from an oxidative stress other than glucose. These results underline the possibility that an improvement in the redox state of SH-SY5Y cells by Trx-mimetic peptides could decrease TXNIP/TBP-2 transcription.

### CB3 activates AMPK

The involvement of AMPK in diabetes was reported in previous studies [31], [49], [32]. AMPK activation promotes glucose uptake and removal from the periphery [50]. Metformin, an activator of AMPK, markedly inhibits TXNIP/TBP-2 mRNA and protein expression. It was suggested to function by inhibiting complex I in the mitochondrial respiratory system, partially via AMPK [51], [31], and through regulation of TXNIP/TBP-2 transcription [49]. AMPK activity is diminished in the muscle and/or liver of ZDF rats. Treatment with the AMPK activator (AICAR) prevented the development of diabetes, and an increase in triglyceride content in liver, muscle and pancreatic islets [52]. Furthermore, pancreatic β-cell morphology was almost normal in AICAR-treated animals, indicating that chronic AMPK activation in vivo might preserve β-cell function [53].

Here we show that in the ZDF brain phosphorylated AMPK was significantly elevated in both CB3- and Rosi-treated rats. To our knowledge, this is the first study demonstrating AMPK activation by a Trx1 mimetic peptide. The increase in AMPK phosphorylation in the brain of CB3 treated ZDF-rats, was in good correlation with a decrease in TXNIP/TBP-2 expression and subsequent blocking of the phosphorylation of p70S6K through the mTOR–p70S6K pathway (Fig. 7).

In the Rosi-treated rats AMPK phosphorylation was significantly increased as expected; however, no apparent reduction in TXNIP/TBP-2 was observed. Further studies are needed to explore why repression of TXNIP/TBP-2 transcription, shown by metformin [31], was not observed in the ZDF brain of Rosi-treated rats.

In summary, in the present 28 days-term experiment the thioredoxin mimetic peptide CB3, attenuated p38^MAPK^ and JNK activity, diminished TXNIP/TBP-2 over expression, and activated AMPK in the brain of ZDF rats. The anti inflammatory effects of CB3 in the brain of the ZDF rat were mediated through the MAPK-AMPK-mTOR-p70^SP6^ signaling pathway. CB3 displayed these protective effects without reducing glucose triglyceride levels or insulin indexes, as opposed to Rosi-treated ZDF rats, in which the decrease in glucose triglyceride levels or insulin indexes account for the decrease in the neuro-inflammatory signaling. The significant decrease in TXNIP/TBP-2 expression in the brain was observed only in the CB3- and not in the Rosi-treated rats. This is the first study that demonstrates significant protective effects by a Trx1 mimetic peptide in the brain of diabetic animals. We suggest that the reduction in the activation of the stress signaling in the brain could lower the risk factor for an accelerated rate of cognitive decline and memory impairments associated with diabetes..

## Contribution

M.C.-K. researched data, contributed discussion, reviewed/edited manuscript; L.K. researched data, reviewed manuscript; M.T. researched data, contributed discussion, reviewed manuscript; H.B. researched data; J.M.L. research data reviewed manuscript T.M. and Y.L. researched data reviewed manuscript; D.A. wrote manuscript and is the guarantor responsible for the study design, access to data, and the decision to submit and publish the manuscript.

## References

[1] Tang J., Pei Y., Zhou G. (2013). When aging-onset diabetes is coming across with Alzheimer disease: comparable pathogenesis and therapy. Exp. Gerontol..

[2] Schubert M., Brazil D.P., Burks D.J., Kushner J.A., Ye J., Flint C.L. (2003). Insulin receptor substrate-2 deficiency impairs brain growth and promotes tau phosphorylation. J. Neurosci..

[3] Holmgren A., Lu J. (2010). Thioredoxin and thioredoxin reductase: current research with special reference to human disease. Biochem. Biophys. Res. Commun..

[4] Arner E.S., Holmgren A. (2000). Physiological functions of thioredoxin and thioredoxin reductase. Eur. J. Biochem..

[5] Saitoh M., Nishitoh H., Fujii M., Takeda K., Tobiume K., Sawada Y. (1998). Mammalian thioredoxin is a direct inhibitor of apoptosis signal-regulating kinase (ASK) 1. EMBO J..

[6] Chung J.W., Jeon J.H., Yoon S.R., Choi I. (2006). Vitamin D3 upregulated protein 1 (VDUP1) is a regulator for redox signaling and stress-mediated diseases. J. Dermatol..

[7] Nishiyama A., Masutani H., Nakamura H., Nishinaka Y., Yodoi J. (2001). Redox regulation by thioredoxin and thioredoxin-binding proteins. IUBMB Life.

[8] Yoshioka J., Schreiter E.R., Lee R.T. (2006). Role of thioredoxin in cell growth through interactions with signaling molecules. Antioxid. Redox Signal..

[9] Junn E., Han S.H., Im J.Y., Yang Y., Cho E.W., Um H.D. (2000). Vitamin D3 up-regulated protein 1 mediates oxidative stress via suppressing the thioredoxin function. J. Immunol..

[10] Kaimul A.M., Nakamura H., Masutani H., Yodoi J. (2007). Thioredoxin and thioredoxin-binding protein-2 in cancer and metabolic syndrome. Free Radic. Biol. Med..

[11] Kim S.Y., Suh H.W., Chung J.W., Yoon S.R., Choi I. (2007). Diverse functions of VDUP1 in cell proliferation, differentiation, and diseases. Cell. Mol. Immunol..

[12] Tonissen K.F., Di Trapani G. (2009). Thioredoxin system inhibitors as mediators of apoptosis for cancer therapy. Mol. Nutr. Food Res..

[13] Yoshihara E., Chen Z., Matsuo Y., Masutani H., Yodoi J. (2010). Thiol redox transitions by thioredoxin and thioredoxin-binding protein-2 in cell signaling. Methods Enzymol..

[14] Saitoh T., Tanaka S., Koike T. (2001). Rapid induction and Ca(2+) influx-mediated suppression of vitamin D3 up-regulated protein 1 (VDUP1) mRNA in cerebellar granule neurons undergoing apoptosis. J. Neurochem..

[15] Kim G.S., Jung J.E., Narasimhan P., Sakata H., Chan P.H. (2012). Induction of thioredoxin-interacting protein is mediated by oxidative stress, calcium, and glucose after brain injury in mice. Neurobiol. Dis..

[16] Mitchelhill K.I., Stapleton D., Gao G., House C., Michell B., Katsis F. (1994). Mammalian AMP-activated protein kinase shares structural and functional homology with the catalytic domain of yeast Snf1 protein kinase. J. Biol. Chem..

[17] Woods A., Cheung P.C., Smith F.C., Davison M.D., Scott J., Beri R.K. (1996). Characterization of AMP-activated protein kinase beta and gamma subunits. Assembly of the heterotrimeric complex in vitro. J. Biol. Chem..

[18] Hwang I.K., Kim I.Y., Joo E.J., Shin J.H., Choi J.W., Won M.H. (2010). Metformin normalizes type 2 diabetes-induced decrease in cell proliferation and neuroblast differentiation in the rat dentate gyrus. Neurochem. Res..

[19] Hurtado-Carneiro V., Sanz C., Roncero I., Vazquez P., Blazquez E., Alvarez E. (2012). Glucagon-like peptide 1 (GLP-1) can reverse AMP-activated protein kinase (AMPK) and S6 kinase (P70S6K) activities induced by fluctuations in glucose levels in hypothalamic areas involved in feeding behaviour. Mol. Neurobiol..

[20] Liu C., Wu J., Zou M.H. (2012). Activation of AMP-activated protein kinase alleviates high-glucose-induced dysfunction of brain microvascular endothelial cell tight-junction dynamics. Free Radic. Biol. Med..

[21] Harmon J.S., Gleason C.E., Tanaka Y., Oseid E.A., Hunter-Berger K.K., Robertson RP. (1999). In vivo prevention of hyperglycemia also prevents glucotoxic effects on PDX-1 and insulin gene expression. Diabetes.

[22] Tanaka Y., Gleason C.E., Tran P.O., Harmon J.S., Robertson R.P. (1999). Prevention of glucose toxicity in HIT-T15 cells and Zucker diabetic fatty rats by antioxidants. Proc. Natl. Acad. Sci. USA.

[23] Robertson R.P., Harmon J., Tran P.O, Tanaka Y., Takahashi H. (2003). Glucose toxicity in beta-cells: type 2 diabetes, good radicals gone bad, and the glutathione connection. Diabetes.

[24] Kaneto H., Xu G., Fujii N., Kim S., Bonner-Weir S., Weir G.C. (2002). Involvement of c-Jun N-terminal kinase in oxidative stress-mediated suppression of insulin gene expression. J. Biol. Chem..

[25] Hirosumi J., Tuncman G., Chang L., Gorgun C.Z., Uysal K.T., Maeda K. (2002). A central role for JNK in obesity and insulin resistance. Nature.

[26] Bachnoff N., Trus M., Atlas D. (2011). Alleviation of oxidative stress by potent and selective thioredoxin-mimetic peptides. Free Radic. Biol. Med..

[27] Cohen-Kutner M., Khomsky L., Trus M., Aisner Y., Niv M.Y., Benhar M. (2013). Thioredoxin-mimetic peptides (TXM) reverse auranofin induced apoptosis and restore insulin secretion in insulinoma cells. Biochem. Pharmacol..

[28] Kim S.R., Lee K.S., Park S.J., Min K.H., Lee M.H., Lee K.A. (2011). A novel dithiol amide CB3 attenuates allergic airway disease through negative regulation of p38 mitogen-activated protein kinase. Am. J. Respir. Crit. Care Med..

[29] Bartov O., Sultana R., Butterfield D.A., Atlas D. (2006). Low molecular weight thiol amides attenuate MAPK activity and protect primary neurons from Abeta(1-42) toxicity. Brain Res..

[30] Aikin R., Rosenberg L., Paraskevas S., Maysinger D. (2004). Inhibition of caspase-mediated PARP-1 cleavage results in increased necrosis in isolated islets of Langerhans. J. Mol. Med. (Berl).

[31] Chai T.F., Hong S.Y., He H., Zheng L., Hagen T., Luo Y. (2012). A potential mechanism of metformin-mediated regulation of glucose homeostasis: inhibition of Thioredoxin-interacting protein (Txnip) gene expression. Cell Signal..

[32] Andres A.M., Ratliff E.P, Sachithanantham S., Hui S.T. (2011). Diminished AMPK signaling response to fasting in thioredoxin-interacting protein knockout mice. FEBS Lett..

[33] Harper S.J., LoGrasso P. (2001). Signalling for survival and death in neurones: the role of stress-activated kinases, JNK and p38. Cell Signal..

[34] Geraldes P., Hiraoka-Yamamoto J., Matsumoto M., Clermont A., Leitges M., Marette A. (2009). Activation of PKC-delta and SHP-1 by hyperglycemia causes vascular cell apoptosis and diabetic retinopathy. Nat. Med..

[35] Matsuzawa A., Nishitoh H., Tobiume K., Takeda K., Ichijo H. (2002). Physiological roles of ASK1-mediated signal transduction in oxidative stress- and endoplasmic reticulum stress-induced apoptosis: advanced findings from ASK1 knockout mice. Antioxid. Redox Signal..

[36] Shalev A., Pise-Masison C.A., Radonovich M., Hoffmann S.C., Hirshberg B., Brady J.N. (2002). Oligonucleotide microarray analysis of intact human pancreatic islets: identification of glucose-responsive genes and a highly regulated TGFbeta signaling pathway. Endocrinology.

[37] Hardie D.G., Ross F.A., Hawley S.A. (2012). AMPK: a nutrient and energy sensor that maintains energy homeostasis. Nat. Rev. Mol. Cell Biol..

[38] Decker P., Muller S. (2002). Modulating poly (ADP-ribose) polymerase activity: potential for the prevention and therapy of pathogenic situations involving DNA damage and oxidative stress. Curr. Pharm. Biotechnol..

[39] Freeman L.R, Zhang L., Nair A., Dasuri K., Francis J., Fernandez-Kim S.O. (2013). Obesity increases cerebrocortical reactive oxygen species and impairs brainfunction. Free Radic. Biol. Med..

[40] Ohara T., Doi Y., Ninomiya T., Hirakawa Y., Hata J., Iwaki T. (2011). Glucose tolerance status and risk of dementia in the community: the Hisayama study. Neurology.

[41] Kalmijn S., Launer L.J., Ott A., Witteman J.C., Hofman A., Breteler M.M. (1997). Dietary fat intake and the risk of incident dementia in the Rotterdam sudy. Ann. Neurol..

[42] White C.L., Pistell P.J., Purpera M.N., Gupta S., Fernandez-Kim S.O., Hise T.L. (2009). Effects of high fat diet on Morris maze performance, oxidative stress, and inflammation in rats: contributions of maternal diet. Neurobiol. Dis..

[43] Tanaka Y., Tran P.O., Harmon J., Robertson R.P. (2002). A role for glutathione peroxidase in protecting pancreatic beta cells against oxidative stress in a model of glucose toxicity. Proc. Natl. Acad. Sci. USA.

[44] Wang Y., De Keulenaer G.W., Lee R.T. (2002). Vitamin D(3)-up-regulated protein-1 is a stress-responsive gene that regulates cardiomyocyte viability through interaction with thioredoxin. J. Biol. Chem..

[45] Chen J., Fontes G., Saxena G., Poitout V., Shalev A. (2010). Lack of TXNIP protects against mitochondria-mediated apoptosis but not against fatty acid-induced ER stress-mediated beta-cell death. Diabetes.

[46] Parikh H., Carlsson E., Chutkow W.A., Johansson L.E., Storgaard H., Poulsen P. (2007). TXNIP regulates peripheral glucose metabolism in humans. PLoS Med..

[47] Takagi Y., Mitsui A., Nishiyama A., Nozaki K., Sono H., Gon Y. (1999). Overexpression of thioredoxin in transgenic mice attenuates focal ischemic brain damage. Proc. Natl. Acad. Sci. USA.

[48] Chen J., Saxena G., Mungrue I.N., Lusis A.J., Shalev A. (2008). Thioredoxin-interacting protein: a critical link between glucose toxicity and beta-cell apoptosis. Diabetes.

[49] Shaked M., Ketzinel-Gilad M., Cerasi E., Kaiser N., Leibowitz G. (2012). AMP-activated protein kinase (AMPK) mediates nutrient regulation of thioredoxin-interacting protein (TXNIP) in pancreatic beta-cells. PLoS One.

[50] Marsin A.S., Bertrand L., Rider M.H., Deprez J., Beauloye C., Vincent M.F. (2000). Phosphorylation and activation of heart PFK-2 by AMPK has a role in the stimulation of glycolysis during ischaemia. Curr. Biol..

[51] Zhou G., Myers R., Li Y., Chen Y., Shen X., Fenyk-Melody J. (2001). Role of AMP-activated protein kinase in mechanism of metformin action. J. Clin. Invest.

[52] Yu X., McCorkle S., Wang M., Lee Y., Li J., Saha A.K. (2004). Leptinomimetic effects of the AMP kinase activator AICAR in leptin-resistant rats: prevention of diabetes and ectopic lipid deposition. Diabetologia.

[53] Pold R., Jensen L.S., Jessen N., Buhl E.S., Schmitz O., Flyvbjerg A. (2005). Long-term AICAR administration and exercise prevents diabetes in ZDF rats. Diabetes.

